# Transforming a Master of Public Health Program to Address Public Health Practice Needs

**Published:** 2005-12-15

**Authors:** Lynn D Woodhouse, Alberto C Cardelle, Steven W Godin, Steven E Shive, Tonya L Williams, Adenike C Bitto, Elizabeth A Brensinger

**Affiliations:** East Stroudsburg University; Health Services Undergraduate Program, Health Department; Health Department; Health Department; Health Department; Health Department, East Stroudsburg University, East Stroudsburg, Pa; East Stroudsburg University, East Stroudsburg, Pa, and Consultant, RedRoad Enterprises, New Tripoli, Pa

## Abstract

The future of the constantly changing public health profession is tied to the development of practice skills through competency-based training. In this article, we describe a program change in the Master of Public Health program at East Stroudsburg University in northeastern Pennsylvania. The first goal of the program transition was to ensure that all program elements included the relevant vision, values, mission, goals, and objectives. The second goal was to use continuous data input and evaluation to incorporate opportunities for flexible assessments. The change process helped the university faculty define the program's vision and fostered an environment of community collaboration that guides training for public health professionals.

## Introduction

Public health is an interdisciplinary profession undergoing dynamic but sometimes conflicting changes. The future of public health can be enhanced by emphasizing the development of practice skills (1-5).

To increase the effectiveness of the public health profession and progress toward the goal of having the healthiest possible population, various adaptable approaches for improving graduate public health training and the skills of the public health workforce continue to emerge (4,6-9). It is important that emerging approaches to public health training support the development of competency-based training grounded in curriculum models (2). It is equally important that the training programs support the broad vision of ensuring social justice and promoting the elimination of health disparities.

East Stroudsburg University (ESU) is one of 14 institutions in the Pennsylvania State System of Higher Learning. Faculty members in ESU's accredited Master of Public Health (MPH) program have a documented history of improving the quality of its curriculum (7). The recent program change described in this article provides a potential model for refocusing graduate public health programs on community health and highlighting community-health education and practice. Given the need for quality assurance, the emphasis on outcomes, and the competition for social jurisdiction among overlapping professions (8), this model of change may help other programs work toward similar goals.

## The Change Process

To ensure that the accredited MPH program at ESU continued to meet the public health needs of our communities and region (northeastern Pennsylvania) in a rapidly changing environment, faculty members who taught required public health courses (the ESU MPH Public Health Faculty Council) in 2003 began developing an adaptable model for change. With input from students, graduates, and community public health professionals, we began a multistage, interactive, community-focused process to transform our graduate public health education training program. The new curriculum was approved in fall 2004.

The program transition was based on a feedback system that highlighted the need for change at every program level. The first goal was to ensure that all program elements incorporated the appropriate program vision, values, mission, goals, and objectives, or VVMGO. The faculty members decided that the program VVMGO should focus on social justice and community health from an ecological perspective rather than focus on promoting program success (3,10,11). The second goal was to incorporate opportunities for flexible assessments through continuous data input and evaluation at every level. We envisioned a process that would blend the adaptability needed to use data with our efforts to keep the program focused on community health.

The figure shows the continual feedback process. The faculty began the process in 2003 with *point 1;* however, other graduate programs could begin using the model at any entry point.

**Figure:**
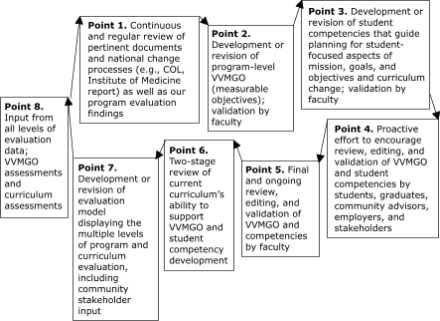
Feedback loop for the initial and continual development processes for program vision, values, mission, goals, and objectives (VVMGO); student competencies; and the curriculum learning objectives and assessments linked to student competencies. The model was adapted from iterations of planning materials used from 2003 to 2005. COL indicates Council on Linkages Between Academia and Public Health Practice.

During the 2-year process, five national initiatives affecting the public health workforce influenced our activities: 1) The Institute of Medicine (IOM) report, “Who Will Keep the Public Healthy? Educating the Public Health Workforce for the 21st Century,” which emphasizes practice experience, the ecological model, and expanding the core curriculum of public health (3); 2) the impact of a more than 10-year dialogue about developing a process for credentialing the public health workforce (2,12); 3) the development of multiple sets of public health competencies from many stakeholders (2,3,13-16); 4) the existing graduate roles and competencies for health education (16, 17); and 5) the current and future requirements for public health program accreditation, including the Council on Education for Public Health (CEPH) accreditation criteria and efforts to blend the MPH in health education concentration and the health education approval and accreditation processes at the graduate and undergraduate levels (18,19).

Four interactive stages of development emerged from an ongoing strategic planning process. The first stage, based on a synthesis of the previously mentioned materials and an examination of regional needs, involved developing a revised draft proposing new program VVMGO. Once approved by the faculty, the draft of the program VVMGO was used to guide the second stage. (A complete list of the revised ESU MPH program VVMGO is available from www.esu.edu/mph)

The second stage involved developing a list of student competencies that were grounded in the ecological model (3,11) and organized into 10 domains. These competencies were compiled after an interactive process involving students, graduates, community stakeholders, employers, community advisors, and faculty members (Figure, *points 1* to *4*). (A complete list of the revised ESU MPH program student competencies is available from www.esu.edu/mph.) The domains and competencies that emerged are primarily a combination of competency lists from the Centers for Disease Control and Prevention (CDC) Public Health Prevention Service, the Council on Linkages Between Academia and Public Health Practice, the Columbia University School of Nursing, and the Joint Committee for the Development of Graduate-Level Preparation Standards, as well as competencies from many general or discipline-specific approaches provided in the references of the 2003 IOM report and on the CDC Web site (2,3). These competency frameworks were continually synthesized, considering content knowledge and skill development required in each framework. Using a matrix or chart as a guide, the third stage included an examination of each course and required program activity to determine the relevance of selected competencies for the program curriculum. While focusing on the VVMGO, the fourth stage involved examining each relevant competency to determine whether it was a current focus of the program and if so, how it was being assessed, or whether it should become a focus and if so, how it should be emphasized.

In August and September 2003, the two written drafts were shared with students, graduates, and community public health professionals. The drafts were accompanied by a cover letter describing our interactive process and requesting input, validation, or both (Figure, *point 5*). Participants ranked the importance of goals, objectives, and competencies and suggested changes, validated the draft, or both. We allowed this feedback to be anonymous (although many people signed their submissions), and the information was returned in our envelope.

Aided by mailed-in information and data from 4 years of program evaluation findings (2001–2004), including student and graduate surveys about curriculum value and outcomes of the program, exit-interview summaries, and internship preceptor interviews, we began the process of revising the curriculum. The revision focused on the courses, student assessments, and program requirements, with the goal of ensuring that graduates who completed the program would have the identified competencies (Figure, *point 6*). Learning objectives for several courses were changed, experiential learning was expanded, and new courses were added.

## Integrated Evaluation Processes

To ensure that program changes were monitored effectively, a modified logic model was created to illustrate the relationships among the revised curriculum requirements, revised course learning objectives, and the program's VVMGO and student competencies (Table). The model was developed to help plan the processes for and implementation of the new evaluation, and its development will help us monitor the program's outcomes and allow the faculty to revise the evaluation of the program as needed (Figure, *point 7*). Creating a visual representation of the interactive nature of the program helped the faculty embrace the idea that program success in all areas is necessary to enhance community health and community health practice.

As part of a CEPH reaccreditation self-study and site visits in spring and fall 2004, meetings were conducted with community advisors, stakeholders, students, graduates, and community public health professionals to obtain  additional input into and final validation of the proposed changes. In addition, the first round of evaluation data using the new processes enhanced this assessment (Figure, *point 8*). Although the revised VVMGO, competencies, curriculum, and evaluation plan have only been implemented for a year, the evaluation — including a revised student and graduate survey, revised outcome measures, and a greater emphasis on community stakeholder input — has been providing preliminary information. Some successes have been revealed, as have areas that need more emphasis, such as environmental health, in which we need to expand experiences and internship opportunities.

## Value of the Process

An important product of this holistic process of program change is the impact on the faculty council. Because the change process was grounded in strategic planning, it helped us define what the faculty and the program should be able to accomplish. It also fostered a culture — a shared vision — of community collaboration that guides the training for public health education practice, applied social behavioral science research, and population-based initiatives emerging from the program's students, graduates, and faculty members (9,20-23). This vision may be atypical for some graduate public health training institutions, but we consider it an important component of a high-quality graduate public health training experience (22).

Many alternative approaches can be used to ensure that the VVMGO of a graduate public health training program support and guide the students, graduates, and faculty members and facilitate community efforts to enhance the health of the public. The ESU MPH program model process was successful for the MPH program. In the future, the process will serve as a quality-control mechanism for the evolution of public health worker certification or credentialing. The next step is to use the process to ensure that the ESU undergraduate community-health and health-services programs are effectively linked to the graduate program and can meet public health workforce needs by graduating students with core public health skills, health-services skills, community-health practice and education skills, or all of these. The emerging potential for undergraduate public health program accreditation makes this step essential.

## Figures and Tables

**Table:** Relationships Among VVMGO, Student Competencies, and Curriculum, East Stroudsburg University Master of Public Health Program^a^

**Program Mission^b^**	**Program Goals**	**Program Objectives and Measures**	**Student Competency Domains^d^**
To enhance the quality of human health through the practice of population-based health education that is responsive to the dynamic, ecologically based, interdependent nature of human systems and the environment	To provide leadership and service	Provide expertise to and serve as consultants for public health organizations Collaborate with public and private health agencies Provide leadership to public health and community-health education organizations	*Analytic assessment and epidemiology:* seven courses and requirements linked to this domain
	To promote research	Promote a global research agenda^c^ Increase opportunities for community-based participatory research^c^ Stimulate ecologically based interdisciplinary research Facilitate dissemination and publication of faculty research in their field of expertise Promote ethical research, according to National Institutes of Health and Institutional Review Board requirements^c^ Develop research skills^c^	*Basic public health sciences:* seven courses and requirements linked to this domain *Policy and planning:* three courses and requirements linked to this domain
	To strengthen public education health practice	Have regular advisory and continuing education activities and meetings Develop continuing education programs to meet needs identified in meetings	*Communication:* three courses and requirements linked to this domain
	To prepare graduates who can improve public health practice	Develop competency in all domains; maintain 3.0 grade point average in all requirements and course work^c^ Develop research competencies and produce publishable articles^c^ Develop ability to present research orally; present research article orally^c^ Successfully complete oral examination and internship^c^ Report favorably on support services; obtain complete student and graduate surveys^c^	*Cultural competency:* three courses and requirements linked to this domain *Community practice:* five courses and requirements linked to this domain *Financial and management:* two courses linked to this domain *Health services organization:* two courses linked to this domain
	To produce graduates who are leaders in the field	Maintain diverse student body Maintain student body with diverse educational backgrounds Assist with graduate employment; maintain graduate database Document graduate outcomes through student survey^c^ Document faculty and student service in database^c^	*Leadership and professionalism:* four courses and requirements linked to this domain *Preparedness:* two courses linked to this domain

a VVMGO indicates vision, values, mission, goals, and objectives.

b Mission, vision, and values must be supported by goals and objectives. (A complete list of VVMGO is available from www.esu.edu/mph.)

c Assessment of student or graduate competency development is linked to this program objective. (A complete list of student competencies and required courses in which competency development is assessed is available from www.esu.edu/mph.)

d Courses in which course learning objectives and assessments are linked to competencies in each domain.
